# 

**Published:** 1887-03

**Authors:** 


CONTENTS

Vol. 34 No. 3.

PAGE.

Somniloquism ..... 49
Oxygen—Its Agency in Therapeutics ..... 53
Woodlands and Malaria ..... 56
Sore Eyes ..... 60
Horsford's Acid Phosphate ..... 61
The Skin, its Correlations and Functions ..... 62
The Heart, its Functions ..... 63
Dream Land, Still they Come ..... 64
Singular Pre-Natal Affliction ..... 66
Book Notices ..... 67
Household ..... 69
Miscellaneous ..... 71

PAGE.

INDEX TO ADVERTISEMENTS OF HALF A PAGE AND OVER.

Winchester's Hypophosphate .. I
Lactated Food ..... II
Celerina ..... III
Lactopeptine ..... IV
Castoria ..... IV
Horsford's Acid Phosphate ..... V
Caulocorea ..... V
Empire Syringe Co. ..... VI
Bovinine ..... VII
Ten Valuable Books Free ..... VIII
Hollow Suppositories, 2d page cover
Dr. Carter Moffats' Ammoniaphone, ..... 3d page cover
Goodyear Rubber Co. 3d page cover

